# Municipal distribution of ovarian cancer mortality in Spain

**DOI:** 10.1186/1471-2407-8-258

**Published:** 2008-09-12

**Authors:** Virginia Lope, Marina Pollán, Beatriz Pérez-Gómez, Nuria Aragonés, Enrique Vidal, Diana Gómez-Barroso, Rebeca Ramis, Javier García-Pérez, Anna Cabanes, Gonzalo López-Abente

**Affiliations:** 1 CIBER en Epidemiología y Salud Pública (CIBERESP), Madrid, Spain; 2 Cancer and Environmental Epidemiology Unit, National Centre for Epidemiology, Carlos III Institute of Health, Madrid, Spain; 3 Public Health Surveillance Unit. National Centre for Epidemiology, Carlos III Institute of Health, Madrid, Spain

## Abstract

**Background:**

Spain was the country that registered the greatest increases in ovarian cancer mortality in Europe. This study describes the municipal distribution of ovarian cancer mortality in Spain using spatial models for small-area analysis.

**Methods:**

Smoothed relative risks of ovarian cancer mortality were obtained, using the Besag, York and Molliè autoregressive spatial model. Standardised mortality ratios, smoothed relative risks, and distribution of the posterior probability of relative risks being greater than 1 were depicted on municipal maps.

**Results:**

During the study period (1989–1998), 13,869 ovarian cancer deaths were registered in 2,718 Spanish towns, accounting for 4% of all cancer-related deaths among women. The highest relative risks were mainly concentrated in three areas, i.e., the interior of Barcelona and Gerona (north-east Spain), the north of Lugo and Asturias (north-west Spain) and along the Seville-Huelva boundary (in the south-west). Eivissa (Balearic Islands) and El Hierro (Canary Islands) also registered increased risks.

**Conclusion:**

Well established ovarian cancer risk factors might not contribute significantly to the municipal distribution of ovarian cancer mortality. Environmental and occupational exposures possibly linked to this pattern and prevalent in specific regions, are discussed in this paper. Small-area geographical studies are effective instruments for detecting risk areas that may otherwise remain concealed on a more reduced scale.

## Background

In 2002, ovarian cancer was the sixth leading tumour and the seventh leading cause of cancer-related death in women world-wide [1]. In Spain, a total of 1760 ovarian cancer deaths were registered in 2006, accounting for 4.6% of all cancer-related deaths in women [2]. Insofar as incidence is concerned, is highest in developed countries. Within Europe, the lowest rates correspond to Mediterranean countries whereas the highest are found in Northern Europe. In 2002, the Spanish incidence rate, adjusted for the standard world population, was estimated at 9.9 cases per 100,000 person-years [3]. However, data of cancer incidence obtained from the provincial registers available in Spain differ from 11 cases per 100,000 person-years in Asturias (in the period 1996–2000) to 6 cases in Albacete (in the period 1998–2001) [4]. In terms of trend, Spain witnessed an increase of 2% p.a. in this tumour's incidence from the late 1980s to the late 1990s [5]. On the other hand, Spain along with Greece, is the country to have recorded the greatest increases in mortality due to this tumour in Europe [6]. Analysis of the components of this trend reveals a notably strong and sustained increase in birth-cohort-related risk until the 1930s, with the slope levelling off thereafter. A moderate period effect is also in evidence, with risk rising from 1972–1976 onwards [7]. In Spain, survival from this tumour in the period 1990–1994 ranked among the highest in Europe, with a cumulative survival of 43% at 5 years. This 5-year survival is substantially lower for females aged 75 and over (25%) compared with younger females (73% 15–44 years) [8], and it has not changed in a ten year period showing a rate of 35.8% in 1990–94 and a rate of 36.5% in 1995–99 [9].

Well-confirmed risk factors for ovarian cancer are age (the tumour being rare in women under 40 years, with the risk increasing sharply thereafter), family history of ovarian cancer, BRCA carrier status and endometriosis, whereas increasing parity, oral contraceptive use, and tubal ligation are inversely associated with risk. There is also a probable increase in risk with postmenopausal hormone therapy, ionising radiation, asbestos exposures and talc use, and a decrease in risk with lactation and hysterectomy [10-12]. There are a number of hypotheses as to the aetiology of ovarian cancer. Of these, the most cited is the incessant ovulation hypothesis [13], which states that long duration of ovulatory menstrual cycles increases the risk of developing ovarian cancer. However, there are other alternative theories to explain the pathogenesis of this tumour, such as the inflammation hypothesis [14], the retrograde transport hypothesis [15] and several hormonal theories, including the gonadotropin, androgen, progesterone, oestrogen, insulin-like growth factor-I and insulin hypotheses [16].

One of the classic approaches in epidemiology is the study of geographical distribution. In administrative terms, Spain is divided into Autonomous Regions known as *Comunidades Autónomas*. These are in turn subdivided into provinces and, at the lowest level, into municipalities. Ovarian cancer mortality has been previously studied at a provincial level and has been shown to be more pronounced in the provinces of Asturias, Seville and Barcelona [17]. Small-area analysis allows for a greater level of disaggregation, thereby improving interpretation of results and detection of local effects that might be linked to specific geographic, social or environmental characteristics, while at the same time reducing ecological biases [18]. This study set out: to analyse the spatial distribution of ovarian cancer mortality at a municipal level in Spain, with the aim of highlighting interregional or municipal patterns; and to discuss the possible relationship between such distribution and the risk factors outlined above.

## Methods

Individual entries for the period 1989–1998, corresponding to deaths in towns and cities throughout Spain due to ovarian cancer (International Classification of Diseases, ICD-9 code 183), were used as the case source. These data were supplied by the National Statistics Institute (*Instituto Nacional de Estadística*) for the production of a municipal cancer mortality atlas.

Municipal populations, broken down by age group (18 groups) and sex, were drawn from the 1991 census and 1996 municipal voters roll. These years correspond to the midway points of the two quinquennia comprising the study period (1989–1993 and 1994–1998). The person-years for each five-year period were estimated by multiplying these populations by 5.

Standardised mortality ratios (SMRs) were obtained as the ratio between observed and expected deaths. For the calculation of expected cases, the overall Spanish mortality rates for the above two 5-year periods were applied to each town's person-years by age group, sex and quinquennium.

To draw up these maps, smoothed municipal relative risks (RRs) were calculated using the autoregressive conditional model proposed by Besag, York and Molliè. This model was introduced by Clayton and Kaldor [19], developed by Besag, York and Molliè [20], and subsequently applied in the field of ecological studies [21]. Such models are based on fitting spatial Poisson models with two random-effect terms that take the following into account: a) the effects which vary in a structured manner in space (municipal contiguity); and, b) a component that models the effects which vary among municipalities in an unstructured manner (municipal heterogeneity) [22]. The model takes the following form

$$\begin{array}{l} O_{i}\sim Po(E_{i}\lambda_{i})\\ \log(\lambda_{i})=\alpha +h_{i}+b_{i} \end{array}$$

where: λ_i _is the relative risk in area i; O_i _is the number of deaths in area i; *α *is the intercept; E_i _are the expected number of cases; h_i _is the municipal heterogeneity term; and b_i _is the spatial term.

The models were fitted using Bayesian Markov chain Monte Carlo simulation methods with improper priors [23]. Posterior distributions of RRs were obtained using WinBugs [24]. The criterion of contiguity needed for the model was adjacency of municipal boundaries. Convergence of the simulations was verified using the BOA (Bayesian Output Analysis) R programme library [25]. Given the great number of parameters of the models, the convergence analysis was performed on a randomly selected sample of 10 towns and cities, taking 4 strata defined by municipal size. Convergence of the estimators was achieved before 100,000 iterations. In the present study, a "burn-in" (iterations discarded to ensure convergence) of 300,000 iterations was performed, and the posterior distribution was derived using 5,000 iterations. The CPU time on a Pentium 2 GHz was 18 hours.

A Geographic Information System (GIS) was used to create municipal maps of SMRs, smoothed RR estimates, and the posterior probability that RR > 1. In the case of this last-mentioned indicator, we applied Richardson's criterion [18], which recommends that probabilities over 0.8 be deemed significant.

## Results

From 1989 to 1998, a total of 13,869 deaths due to ovarian cancer were registered in 2,718 Spanish towns. In comparative terms, this tumour caused 4% of cancer deaths, which amounted to 0.8% of all female deaths in this period.

**Figure 1:**
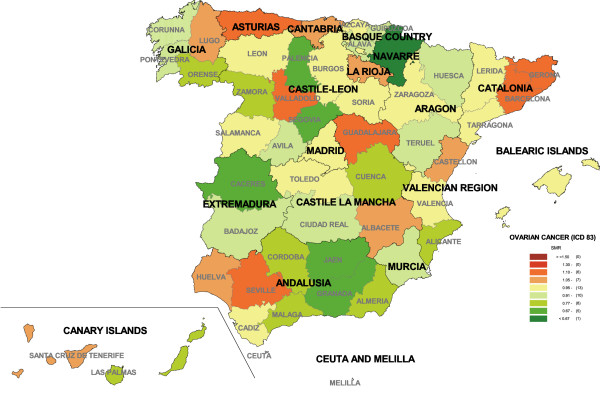
Provincial distribution of ovarian cancer mortality. Spain, 1989–1998.

To give an overall picture, Figure 1 shows ovarian cancer mortality by province. The province with highest mortality was Asturias (SMR = 1.17), followed by Barcelona, Valladolid, Guadalajara, Seville and Gerona. Navarre, in contrast, was the province with lowest mortality (SMR = 0.60).

**Figure 2:**
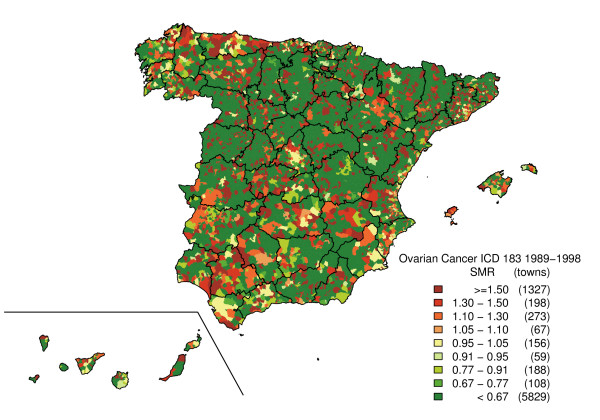
Municipal distribution of ovarian cancer mortality. Standardised mortality ratios (SMR). Spain, 1989–1998.

**Figure 3:**
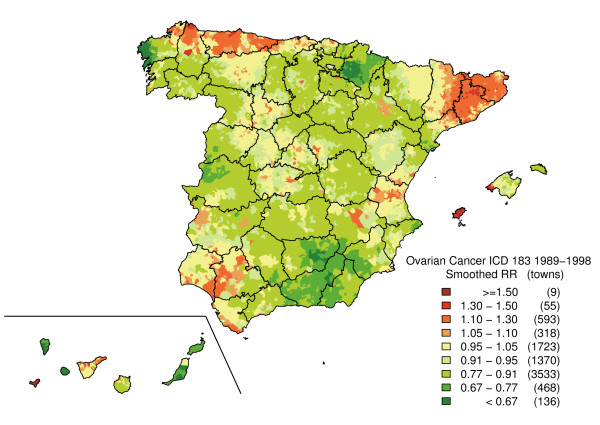
Municipal distribution of ovarian cancer mortality. Smoothed relative risk (RR) under the BYM model. Spain, 1989–1998.

Figure 2 depicts the SMR distribution pattern. This map shows the polarisation of the distribution towards its extremes (towns with and without cases), with no specific pattern being clearly discernable. The "noise" present in this map, deriving from the instability of the indicator, was eliminated by means of the smoothing procedure. From the smoothed map (Figure 3), three major areas will be seen to register the highest risk of ovarian cancer mortality on mainland Spain. The first of these lies in Asturias and the north of Corunna and Lugo (mainly in coastal towns). The second is situated in Catalonia, specifically in the eastern area of Lerida, northern Tarragona, Gerona and Barcelona. In this last-mentioned province, there was a noteworthy concentration of towns with elevated risks in the Osona district. Finally, we detected a third high-risk area formed by towns lying south-east of Huelva, west of Seville and south of Cadiz. With regard to Spain's offshore territories, a higher risk of mortality was observed on Eivissa (Balearic Isles), El Hierro and northern Tenerife (Canary Islands). The regions showing the lowest risk associated with this tumour lay in eastern Andalusia, western Murcia, Navarre, the west of Corunna and on the Canary Islands (except for El Hierro and Tenerife).

**Figure 4:**
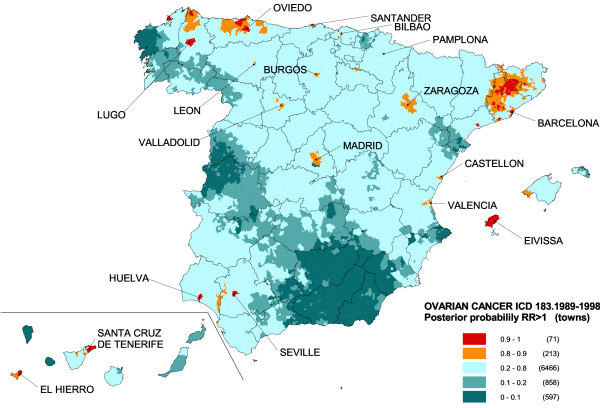
Municipal distribution of ovarian cancer mortality. Posterior probability of RR being greater than 1. Spain, 1989–1998.

On the map depicting the spatial pattern of the posterior probability of RR being greater than 1 (Figure 4), towns with a statistically significant excess risk (probabilities of over 0.8) are shown in orange and red. Many of these towns coincide with provincial capitals, e.g., Lugo, Oviedo, Santander, León, Burgos, Bilbao, Pamplona, Valladolid, Madrid, Zaragoza, Barcelona, Castellón, Valencia, Huelva, Seville and Santa Cruz de Tenerife. However, rural areas with significant excess risk were also detected, such as the islands of Eivissa and El Hierro, the north of Lugo, and inland areas of Barcelona and Gerona, with attention again being drawn to the concentration of highest risk in the Osona district.

Shown in Table 1 is information on the most representative towns with excess ovarian cancer mortality. The selection criteria used were towns having: an RR of over 1.2; a posterior probability that RR > 1 being greater or equal to 0.8; and a difference between observed and expected cases of 3 or more. A total of 49 towns, belonging to 13 provinces, met these criteria. Of these towns, 41% were situated in Catalonia, with Valverde, on the Island of El Hierro, followed by Eivissa being the towns with the highest RRs in Spain.

**Table 1:** Selected towns and cities with excess ovarian cancer mortality.* Spain 1989–1998.


**Autonomous Region Province/Island**	Municipality *	**Obs**	**Exp**	**SMR**	**Smoothed RR**	**P (RR > 1)**

ANDALUSIA						
Cadiz	CHIPIONA	10	3.27	3.06	1.49	0.872
Huelva	HINOJOS	5	1.14	4.39	1.26	0.822
Huelva	HUELVA	57	41.76	1.37	1.24	0.926
Seville	PILAS	10	3.25	3.08	1.39	0.892
Seville	SANLUCAR LA MAYOR	8	2.75	2.91	1.28	0.847
ASTURIAS						
Asturias	AVILES	38	31.00	1.23	1.23	0.934
Asturias	CASTRILLON	14	7.28	1.92	1.45	0.975
Asturias	OVIEDO	104	78.66	1.32	1.28	0.994
Asturias	PILOÑA	10	5.19	1.93	1.24	0.836
Asturias	PRAVIA	8	4.84	1.65	1.34	0.913
Asturias	SOTO DEL BARCO	6	2.12	2.84	1.48	0.941
Asturias	TINEO	12	6.46	1.86	1.27	0.873
Asturias	VILLAVICIOSA	13	7.91	1.64	1.27	0.867
BALEARIC ISLANDS						
Balearic Islands	ANDRAITX	6	2.82	2.13	1.39	0.839
Balearic Islands	CALVIA	11	5.22	2.11	1.36	0.861
Balearic Islands	EIVISSA	16	7.85	2.04	1.71	0.993
CANARY ISLANDS						
Sta. Cruz de Tenerife	CANDELARIA	8	3.12	2.56	1.28	0.846
Sta. Cruz de Tenerife	LAGUNA (LA)	39	30.20	1.29	1.21	0.916
Sta. Cruz de Tenerife	REALEJOS (LOS)	15	7.87	1.91	1.37	0.894
Sta. Cruz de Tenerife	SANTA CRUZ DE TENERIFE	77	62.00	1.24	1.22	0.966
Sta. Cruz de Tenerife	VALVERDE	5	1.48	3.38	2.16	0.927
CANTABRIA						
Cantabria	SANTA CRUZ DE BEZANA	6	1.85	3.25	1.32	0.818
CATALONIA						
Barcelona	BARBERA DEL VALLES	13	6.39	2.03	1.36	0.910
Barcelona	IGUALADA	23	12.42	1.85	1.49	0.983
Barcelona	MARTORELL	12	5.86	2.05	1.41	0.942
Barcelona	MOLINS DE REI	12	6.29	1.91	1.28	0.890
Barcelona	PRATS DE LLUÇANES	6	1.12	5.36	1.58	0.976
Barcelona	PUIG-REIG	6	2.31	2.60	1.37	0.945
Barcelona	SANT CELONI	10	4.17	2.40	1.30	0.863
Barcelona	SANT MARTI SARROCA	4	0.99	4.06	1.24	0.826
Barcelona	SANT PERE DE RIBES	12	4.23	2.84	1.48	0.930
Barcelona	SANT PERE DE RIUDEBITLLES	4	0.83	4.85	1.39	0.895
Barcelona	TORELLO	11	4.15	2.65	1.54	0.981
Barcelona	VIC	22	11.76	1.87	1.51	0.993
Gerona	AMER	4	1.00	4.00	1.36	0.901
Gerona	CELRA	4	0.85	4.70	1.34	0.852
Gerona	PUIGCERDA	7	2.28	3.07	1.53	0.912
Gerona	SALT	11	6.71	1.64	1.31	0.898
Gerona	SANT FELIU DE GUIXOLS	10	6.18	1.62	1.30	0.837
Gerona	SARRIA DE TER	5	1.18	4.25	1.42	0.894
Gerona	TORROELLA DE MONTGRI	6	2.49	2.41	1.29	0.821
Tarragona	REUS	45	29.63	1.52	1.30	0.947
GALICIA						
Corunna	FERROL	44	34.78	1.27	1.24	0.925
Lugo	CERVO	7	3.62	1.94	1.36	0.886
Lugo	LUGO	51	33.02	1.55	1.30	0.945
Lugo	MONDOÑEDO	7	3.37	2.08	1.24	0.846
Lugo	VIVEIRO	12	6.39	1.88	1.42	0.941
VALENCIAN REGION						
Valencia	ALDAIA	12	6.23	1.93	1.26	0.824
Valencia	TORRENT	29	17.13	1.69	1.29	0.897

Obs = observed deaths; Exp = expected deaths; SMR = standard mortality ratio; Smoothed RR = smoothed relative risk; P (RR > 1) = posterior probability that RR > 1.

* Municipalities that fulfil the following criteria: RR > = 1.2; (P(RR) > 1) > = 0.8 and (Obs – Exp) > = 3

## Discussion

This study reveals some differences in the distribution of ovarian cancer mortality at a small-area level in Spain. The towns with highest risk are located in the north of Lugo and Asturias, interior of Barcelona and Gerona, along the Seville-Huelva boundary, and on the islands of Eivissa and El Hierro. Although this pattern is, in general, in line with a study previously undertaken at a provincial level [17], analysis at a municipal level has enabled small areas of higher risk that remained concealed at the provincial scale, to be detected.

Standard errors of SMR are inversely dependent on the number of expected cases. This implies that the most extreme SMRs occur in small population areas and are based on a small number of cases. Maps of unsmoothed SMRs are therefore dominated by "green areas" (low RRs) and "red areas" (high RRs), most likely reflecting random variation. The smoothed map, however, tends to eliminate part of this random variability, solely highlighting those areas in which the risk is concentrated, so that a town with no ovarian cancer deaths could be regarded as having excess risk due to the elevated risk of its neighbours and vice-versa. As a result, this could give rise to false positives (enhancing the risk of some towns) or false negatives (attenuating the risk of others).

When it comes to interpreting results, some factors must be taken into account. Firstly, ovarian carcinoma is an under-certified tumour in Spain, with a detection rate of 74% and confirmation rate of 81%, according to Percy's criteria [26]. In fact, until the introduction of CIE-8 many ovarian cancer deaths were erroneous certified [7]. Accuracy of ovary-neoplasm certification seems to vary widely among studies. While some reported cases of death due to ovarian cancer were really due to abdominal or uterine neoplasms, in some studies, ≪unspecified uterus tumours≫ would appear to include some ovarian cancers as well as tumours of the cervix and endometrium [26]. If this misclassification were not uniform throughout the country, the spatial distribution of mortality due to this tumour might be affected by this problem. However, excess deaths due to ovarian cancer are concentrated in a series of specific towns, whereas certification and coding is uniform across municipalities in one autonomous region. Another point to bear in mind are possible regional differences in quality of medical care, reduced access to it, differences in diagnostic and therapeutic techniques, or even random variation, still present even using smoothed estimates.

Geographical differences in the prevalence of some risk factors documented for this tumour could have an influence on the spatial mortality pattern observed in this study. One well-confirmed risk factor is family history. Approximately 10% of epithelial ovarian tumours are hereditary, with *BRCA *mutations being responsible for the majority of cases [11,27,28]. The contribution of these mutations varies among the populations depending on their penetrance and prevalence, with the latter being possibly influenced by founder mutations. In Spain, a study targeting *BRCA1 *and *BRCA2 *mutations in Spanish families with breast/ovarian cancer highlighted a high proportion of variations that appear to be unique to Spaniards, and the existence of recurrent variations associated with the geographical origin of the families [29]. In support of this argument, mention should be made of the coincidence between the ovarian cancer mortality pattern detected in the south-west of the country and the breast cancer mortality pattern detected in the same area among premenopausal women [30], a group in which the hereditary component assumes major force.

Ovarian cancer mortality in Spain described a strongly rising trend in the 20th century. An important factor in the analysis of the components of this trend is a strong and sustained increase in birth-cohort-related risk from the beginning of the series until the 1930s [6,7,31]. This effect reflects an important change in the lifestyle of Spanish women, determined in great measure by socio-cultural changes, the incorporation of women into the job market, longer educational periods, improvements in the quality of life and swift urbanisation. These events have led to modification of certain hormonal and reproductive factors, both positively and negatively associated with ovarian cancer, such as fertility, use of oral contraceptives, tubal ligation and replacement hormone therapy. We have not sufficiently detailed information to confirm if geographical differences in the prevalence of some of these risk factors have an influence in the mortality pattern observed. Nevertheless, some of these changes (e.g., fall in the birth rate, a rise in the age at which women have their first child or reduced breastfeeding) have occurred earlier in urban areas and might have contributed to the increase in mortality observed in many provincial capitals. In this respect, regions in the north of Spain have registered the highest delay of the age of first birth and the lowest fecundity nationwide. Among these, mention should be made of Asturias as the region with fewest children per woman during the preceding 30 years [32,33].

The concentration of higher ovarian cancer mortality in narrowly localised geographical areas, leads one to consider the possible influence of exposure to certain environmental pollutants or occupational exposures. Many municipalities with high excess risk are localised in the province of Barcelona and the town of Pilas in Seville Province, where the economic activity has traditionally been based on textiles, and it is being currently hit very hard by the crisis in the sector. There are a number of papers in the literature that have found an association between this tumour and the textile or leather industries [34-40], and in practically all cases this association was related with exposure to asbestos fibres [34-36,38]. This type of industry uses chrysotile during the fabric-making operations of carding, spinning, weaving, dyeing and finishing, as well as machinery maintenance [41]. In the early 20^th ^century, mechanisation of the process in the Catalonian textile industry was accompanied by the substitution of women for men [42]. One argument that would support this hypothesis is the fact that pleural cancer mortality is also very high in the same areas of the province of Barcelona [43].

Furthermore, unirrigated crop farming and animal husbandry-basically pig farming- are both activities that have been strongly rooted in this area. One of the major problems confronting this region, and the Osona district in particular, is the nitrate pollution of aquifers as a consequence of the excessive dumping of purines. Many towns in these districts have been declared "vulnerable zones to pollution by nitrates from agricultural sources" [44,45], and the drinking water supplied to inhabitants of these municipalities was found to have a nitrate content exceeding legally stipulated levels [46]. In fact, the disputes generated in areas such as Baix Ter and Osona have been formally brought before the European Commission and, ultimately, the Court of Justice of the European Communities [46]. The Guadalquivir basin has also been affected by contamination of its groundwaters. Twenty five percent of its aquifers register nitrate levels in excess of those permitted by law [47]. Although there is no clear evidence of an association between exposure to nitrates and risk of ovarian cancer, a positive association has indeed been detected with consumption of polluted water in a cohort of Iowa women, with the risk rising in response to increases in the nitrate content of the drinking water [48].

## Conclusion

In brief, the geographical study of municipal ovarian cancer mortality shows a marked excess risk in certain towns in the north of Asturias and Lugo, inland areas of Barcelona and Gerona, along the Seville-Huelva boundary, and on the Islands of Eivissa and El Hierro. Even though well established ovarian cancer risk factors do not seem to contribute significantly to the spatial distribution of ovarian cancer mortality, hormonal and reproductive changes that occurred in women's lifestyle earlier in urban areas might have contributed to the increase in mortality observed in many provincial capitals. Likewise, regional variations in medical care; and different diagnostic and therapeutic techniques could have played a role. Finally, environmental or occupational exposures to certain carcinogenic agents might also have influenced on the more localised excess mortality areas. Nevertheless, the descriptive nature of this study and the limited knowledge of ovarian cancer pathogenesis make it difficult to draw any conclusions on the real determinants implicated in the mortality pattern observed.

## Abbreviations

SMR: Standardised mortality ratio; RR: relative risk; GIS: Geographic Information System.

## Competing interests

The authors declare that they have no competing interests.

## Authors' contributions

VL conceived the idea and wrote the first draft of the manuscript to which all authors subsequently contributed. GLA, MP, NA, and BPG were all involved in designing the study. GLA, RR and EV performed the statistical analysis. DGB draw the maps using GIS software. JGP reviewed the information about possible pollutant sources in the areas with high RR. All authors made contribution to statistical analyses and interpretation of results, revised the manuscript for important intellectual content and read and approved the final manuscript.

## Pre-publication history

The pre-publication history for this paper can be accessed here:


